# LFA-1 knockout inhibited the tumor growth and is correlated with treg cells

**DOI:** 10.1186/s12964-023-01238-6

**Published:** 2023-09-18

**Authors:** Ting Niu, Zhengyang Li, Yiting Huang, Yuxiang Ye, Yilong Liu, Zhijin Ye, Lingbi Jiang, Xiaodong He, Lijing Wang, Jiangchao Li

**Affiliations:** Institute of Basic Medical Sciences, School of Life Sciences and Biopharmaceuticals, Guangdong Pharmaceutical University, No. 280 Waihuan Rd. E, Higher Education Mega Center, 510006 Guangzhou, China

**Keywords:** LFA-1, Treg cells, *Apc*^Min/+^ mice, Tumor growth, Immunotherapy

## Abstract

Cancer immunotherapy has been proven to be clinically effective in multiple types of cancers. Lymphocyte function-associated antigen 1 (LFA-1), a member of the integrin family of adhesion molecules, is expressed mainly on αβ T cells. LFA-1 is associated with tumor immune responses, but its exact mechanism remains unknown. Here, two kinds of mice tumor model of LFA-1 knockout (LFA-1^−/−^) mice bearing subcutaneous tumor and *Apc* ^Min/+^;LFA-1^−/−^ mice were used to confirm that LFA-1 knockout resulted in inhibition of tumor growth. Furthermore, it also demonstrated that the numbers of regulatory T cells (Treg cells) in the spleen, blood, mesenteric lymph nodes were decreased in LFA-1^−/−^ mice, and the numbers of Treg cells in mesenteric lymph nodes were also decreased in *Apc* ^Min/+^;LFA-1^−/−^ mice compared with *Apc* ^Min/+^ mice. LFA-1 inhibitor (BIRT377) was administered to subcutaneous tumor-bearing LFA-1^+/+^ mice, and the results showed that the tumor growth was inhibited and the number of Treg cells was reduced. The analysis of TIMER tumor database indicated that LFA-1 expression is positively associated with Treg cells and TNM stage. Conclusively, this suggests that LFA-1 knockout would inhibit tumor growth and is correlated with Treg cells. LFA-1 may be one potential target for cancer immunotherapy.

Video Abstract

**Supplementary Information:**

The online version contains supplementary material available at 10.1186/s12964-023-01238-6.

## Introduction

Lymphocyte function-associated antigen 1 (LFA-1), also known as *Itgal*, is composed of an α_L_ subunit (CD11a) and a β_2_ subunit (CD18), which form a heterodimer expressed on the surface of all leukocytes, including T cells, B cells, macrophages, and neutrophils [1]. Previous studies have shown that LFA-1 affects hematologic malignancies or tumor invasion and is involved in angiogenesis, leukocyte recruitment, and migration [2, 3]. However, the role of LFA-1 in tumor development and growth remains unclear. Studies showed that the severity of experimental autoimmune encephalomyelitis (EAE) increased after wild-type T cells were transplanted into LFA-1^−/−^ recipient mice with encephalitis and that the Treg cell count in the spleen and thymus of these mice were lower than those in LFA-1^+/+^ mice, which indicated that the development of Treg cells was altered. LFA-1 has both proinflammatory and anti-inflammatory roles in the pathogenesis of EAE [4, 5]. The exacerbation of EAE progression in LFA-1^−/−^ mice may be due to impaired inhibition of autoantigen-specific effector T cells by Treg cells, which present a normal Cytoxic T-cell lymphocyte (CTL) response to viral infection but fail to reject immunogenic tumors grafted into the footpads [5, 6].

Some studies suggested that Treg cells could be a target for cancer therapy [7, 8], and play a key role in preventing autoimmunity, limiting immunopathology, and maintaining immune homeostasis. LFA-1 participates in the recruitment, adhesion, and migration of various immune cells [9, 10], activities that are especially necessary for the transport of T cells to mesenteric lymph nodes and the homing of effector T cells to the colon, which causes chronic intestinal inflammation in the colon [11].

Familial adenomatous polyposis and colorectal cancer are autosomal dominant diseases caused by genetic mutation of the adenomatous polyposis *(APC)* gene located on chromosome 5p21. Mutation occurs at many different sites of APC gene [12–14]. Treg cells were shown to delay the growth of intestinal tumors in *APC* ^Min/+^ mice by suppressing intestinal inflammation [15–17]. *Apc* ^Min/+^ mice exhibit stable heredity and spontaneous development of intestinal adenomas and have been extensively used in tumor immunity and cancer drug discovery.

In our study, we found that LFA-1 knockout suppresses the growth of intestinal tumors in *Apc* ^Min/+^ mice and subcutaneous xenograft tumor growth in LFA-1^−/−^ mice, and the Treg cells were decreased in spleen and blood in mice. BIRT377 is an LFA-1 noncompetitive inhibitor targeting the active conformation of a transmembrane β-2 adhesion receptor that reduces the expression of LFA-1 on white blood cells [18]. BIRT377, which selectively inhibits LFA-1/ICAM-1 mediated binding events in vitro and in vivo, blocks the interaction between T cells and antigen-presenting cells (referred to as an immune synapse) [19]. BIRT377 was used to treat subcutaneous tumor-bearing LFA-1^+/+^ mice, and it can effectively slow down the growth of tumors and down-regulate Treg cells.

In summary, the regulation of Treg cells by LFA-1 knockout might be the main reason for inhibiting tumor growth. Characterization and identification of the function of LFA-1 would facilitate the restoration of immune function in cancer patients and even contribute to the clinical potential of immunotherapy.

## Materials and methods

### Mice

The *APC* transgenic mice and *Apc* ^Min/+^ and LFA-1-deficient mice (LFA-1^−/−^) were purchased from The Jackson Laboratory (C57BL/6J-*Apc* ^Min/+^/J and B6.129S7-Itgal^tm1Bll^/J, respectively). In our study, *Apc* ^Min/+^;LFA-1^−/−^ mice were obtained by crossbreeding *Apc* ^Min/+^ mice with LFA-1^−/−^ mice. After cutting off some toes of mice, tissue DNA was extracted by alkali lysis method, and the genotype was identified by PCR amplification. *Apc* ^Min/+^ primer sequence of gene fragment: Mutant primer: 5’-TTC TGA GAA AGA CAG AAG TTA-3’, Wild-type primer: 5’-TTC CAC TTT GGC ATA AGG C-3’, Common primer: 5’-GCC ATC CCT TCA CGT TAG-3’. LFA-1 mutant primer1: 5’-GCC CTG AAT GAA CTG CAG GAC G-3’, LFA-1 mutant primer2: 5’-CAC GGG TAG CCA ACG CTA TGT C-3’, LFA-1 wild-type primer1: 5’-AGA AGC CAC CAT TTC CCT CT-3’, LFA-1 wild-type primer2: 5’-AGC TGG AGT CCC AGT AGC AA-3’. After carrying out agarose gel electrophoresis on PCR amplification products. The amplified fragment of APC gene mutation primer PCR product is 340 bp, the amplified fragment of APC gene wild-type primer PCR product is 600 bp. *Apc* ^Min/+^ mice are heterozygotes, with mutation bands (340 bp) and wild-type bands (600 bp) double bands in genotype identification. The amplification fragment of LFA-1 gene Mutant primer PCR product is 432 bp, the amplification fragment of LFA-1 gene wild-type primer PCR product is 151 bp. LFA-1^−/−^ mice are homozygous for mutation, and genotyping shows that there are only mutant bands (432 bp) but no wild-type bands (151 bp). *Apc* ^Min/+^;LFA-1^−/−^ mice were screened after the third generation hybridization (Supplemental Fig. 1A). C57BL/6J mice were obtained from the Guangdong Medical Laboratory Animal Center, Guangzhou, China. All mice were housed in the specific pathogen-free barrier facility of the Animal Center of Guangdong Pharmaceutical University (license number SCYK (Guangdong) 2017 − 0125). All mice were sacrificed by dislocating the cervical vertebrae. The Animal Ethics Review Committee approved all Guangdong Pharmaceutical University animal experiments; the number of animal ethics approval was Gdpulac20177006.

### Cell culture

B16F10 melanoma cells (Shanghai Institute of Cell Biology, Chinese Academy of Sciences) were maintained in high-glucose Dulbecco’s modified Eagle’s medium (Invitrogen) containing 10% fetal bovine serum (FBS; GIBCO). The cell was incubated in a humidified chamber containing 5% CO_2_ at 37 °C, and it was confirmed with no mycoplasma contamination by PCR detection.

### Subcutaneous tumor xenograft models

B16F10 melanoma cells (2 × 10^5^ cells per mouse) were injected subcutaneously into LFA-1^−/−^ mice (*n* = 11) and LFA-1^+/+^ mice (*n* = 16) in the lower right groin. Mouse tumor growth and body weight were monitored and recorded once a day. After 2 weeks, these mice were sacrificed, and their blood, tumor tissues, and spleens were harvested and preserved for further analysis.

### Intestinal tumor mouse model

Male *Apc* ^Min/+^ mice and female LFA-1^−/−^ mice aged from 12 to 24 weeks were caged for breeding at a ratio of 1:3. Next, male *Apc* ^Min/+^;LFA-1^+/−^ mice from the first generation were selected for breeding with age-appropriate female LFA-1^−/−^ mice to obtain the second generation. *Apc* ^Min/+^;LFA-1^−/−^ mice from the second generation were identified and were then reared under a hybrid spontaneous intestinal adenoma model for subsequent experimental research (Supplemental Fig. 1A). Survival status and intestinal tumor growth were observed in all *Apc* ^Min/+^;LFA-1^−/−^ mice (*n* = 25) and *Apc* ^Min/+^ mice (*n* = 32). From birth to 48 weeks of age, the *Apc* ^Min/+^;LFA-1^−/−^ mice did not exhibit prominent physiological or histopathological abnormalities, exhibiting only weight loss without a change in the spleen-to-weight ratio (Supplemental Fig. 1B). After the mice were sacrificed, the intestines were harvested, and the length (*L*) and width (*W*) of the tumors were measured. Adenomas were considered to have a diameter > 2 mm; microadenomas, a diameter ≤ 2 mm. The tumor volume was calculated as *V = 0.5×L×W*^*2*^.

### BIRT377 treatment

BIRT377 (Tocris; Cat# 4776) was dissolved in anhydrous ethanol to a concentration of 22.156 mg/mL and stored at 4 ℃. The vehicle was sterile water containing 0.226% ethanol. On the day of injection, the vehicle was heated in a water bath at 37 ℃, and BIRT377 was added to a concentration of 500 ng/20 µL for i.v. injection [20]. Animals were injected within one hour of BIRT377 dilution. B16 tumor-bearing LFA-1^+/+^ mice were divided into two groups. On the 7th day, when the tumors were observed, BIRT377 (20 µL per mouse) was injected through the tail vein into mice in the experimental group (*n* = 13), and mice in the control group (*n* = 10) were injected with 20 µL of vehicle. The total operation time of the injection was less than 2 min without anesthesia. All mice appeared normal following the injection. According to the above methods of injection once a day, 7 days of continuous injection, the mice were euthanized, and the subcutaneous tumors and spleen tissues were then excised for subsequent analysis.

### H&E and IHC

4% Formalin-fixed, paraffin-embedded tissues were prepared. After deparaffinization, 3 μm sections were stained with hematoxylin for 3 min and eosin for 30 s and were then dried and sealed with neutral resin. IHC staining was performed as described on the Abcam website. Antigen retrieval was carried out in citrate buffer (pH 6.0). Sections were incubated with a primary antibody overnight at 4 ℃. Next, sections were incubated with a secondary antibody for 1 h at 37 ℃, and color was developed with DAB (8059 S, CST, USA) chromogenic solution. The primary antibodies included anti-Ki67 (1:100, ab16667, Abcam, USA), anti-CD31 (1:100, ab28364, Abcam, USA), and anti-Foxp3 (1:100, BA2032-1, Boster, China) antibodies. The HRP-conjugated anti-rabbit/mouse secondary antibody was obtained from DAKO. According to the diagnostic guidelines, three pathologists independently scored the HE sections of the intestinal tract tissues. The number of ki67 positive cells was counted and compared. The area of CD31 positive expression was calculated, and the microvascular density (MVD) was compared. Statistical analysis of immunohistochemical staining was performed using *IPP6.0 software*.

### RNA sequencing

The spleens of LFA-1^−/−^ mice (*n* = 4) and LFA-1^+/+^ mice (*n* = 4) were frozen in liquid nitrogen. Total RNA of spleen tissue was extracted by Beijing Genomics Institute (BGI, Wuhan, China) to construct the cDNA library, which was sequenced on the Illumina sequencing platform. The DESeq algorithm was utilized to filter the differentially expressed genes.

### Flow cytometry analysis

FACS analysis was carried out on the blood and tissue samples on a Beckman Coulter Gallios Flow Cytometer. Antibodies specific for the following cell surface markers were used: CD3 conjugated to PerCP-Cy5.5 (clone: 145-2C11), CD4 conjugated to PE (clone: GK1.5), CD8 conjugated to BV421 (clone: 53 − 6.7), and CD25 conjugated to APC (clone: PC61). Moreover, the measurement of intracellular signaling molecules (Alexa Fluor® 488 anti-mouse/rat/human FOXP3 antibody, clone: 150D, BioLegend, USA) was performed for Foxp3 analysis. Each experiment included single-color compensation and an appropriate isotype control, and each sample was independently analyzed at least three times. As evaluated by the mean fluorescence intensity, the percentage of positive cells was determined with FlowJo software (Tree Star, Inc. OR, USA). Lymphocytes were identified by plotting forward vs. side scatter followed by gating on CD3 + T cells, and these cells were then analyzed by separation into CD4 + T and CD8 + T cells. Treg cells were identified as CD25 + Foxp3 + cells gated within CD3 + CD4 + T cells.

### RNA isolation and real-time PCR

Total RNA was extracted from blood, spleens, and mesenteric lymph nodes with TRIzol reagent (Invitrogen, USA) according to the instructions. All reverse transcription reactions were set up using an EVO M-MLV RT Kit (Accurate Biology, China). Real-time quantitative PCR was performed with a SYBR® Green Premix Pro Taq HS qPCR Kit (Accurate Biology, China) on an Applied Biosystems 7500 instrument. The thermal cycling conditions for PCR were as follows: 95 °C for 3 min, followed by 40 cycles at 95 °C for 30 s, 58 °C for 40 s and 72 °C for 50 s, and a final 10 min extension at 72 °C. The PCR primers are listed in Table 1 (Supplementary data).

### Statistical analysis

Data are shown as the mean ± standard deviation (SD) values. The Kaplan–Meier method with the log-rank test were used for survival analysis (GraphPad Prism 5). IHC images were analyzed with *IPP6.0 software*. The Pearson chi-square test was used to compare the IHC scores. Student’s *t* test was used to analyze the real-time PCR results. A *p* value less than 0.05 was considered statistically significant.

## Results

### LFA-1 knockout suppressed tumor growth in mice

**Fig. 1:**
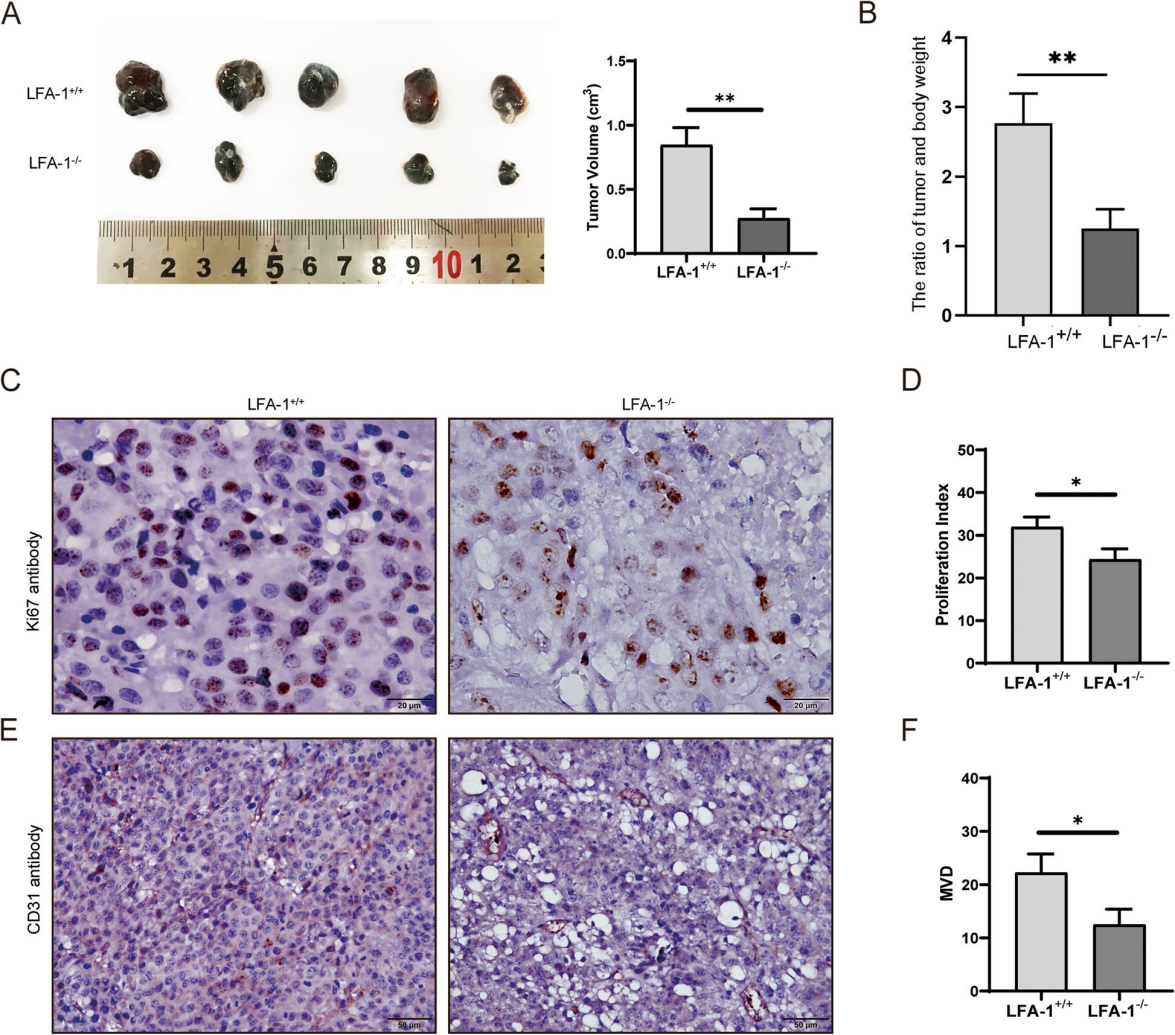
LFA-1 deletion inhibited subcutaneous tumor growth. **A** Comparison and statistics of subcutaneous tumor volume between LFA-1^−/−^ mice (*n* = 7) and LFA-1^+/+^ mice (*n* = 7) (**P < 0.001). **B** LFA-1^−/−^ mice (*n* = 7) and LFA-1^+/+^ mice (*n* = 7) tumor-to-body weight ratio (**P < 0.001). **C** Immunohistochemical staining of Ki67 antibody in subcutaneous melanoma in mice. **D** Proliferation rate in subcutaneous tumors of LFA-1^−/−^ mice (*n* = 3) and LFA-1^+/+^ mice (*n* = 3). **E** Immunohistochemical staining of CD31 antibody in subcutaneous melanoma in mice. **F** The intratumoral microvessel density (MVD) in subcutaneous tumors of LFA-1^−/−^ mice (*n* = 3) and LFA-1^+/+^ mice (*n* = 3)

LFA-1 is a lymphocyte-associated antigen that plays a critical role in inflammation, and regulating immune cells. We established a subcutaneous xenograft tumor model to investigate whether LFA-1 knockout affects tumor growth. Next, the survival rates, body weight, and tumor growth of the mice were recorded after two weeks. The results showed that compared to that in LFA-1^+/+^ +B16 mice (*n* = 7), the tumor volume in LFA-1^−/−^ mice (*n* = 7) was significantly reduced (Fig. 1A**P < 0.01) and the tumor-to-body weight ratio was also reduced between the two groups (Fig. 1B, **P < 0.01). To confirm that pathological processes involved in tumor growth, IHC staining was carried out with anti-Ki67 antibody and anti-CD31 antibody. The results indicated that LFA-1^−/−^ mice exhibited lower tumor cell proliferation and a lower microvascular density (MVD) than control mice (Fig. 1C-F, *P < 0.05), which implies that tumor growth was suppressed in LFA-1 knockout mice.

**Fig. 2:**
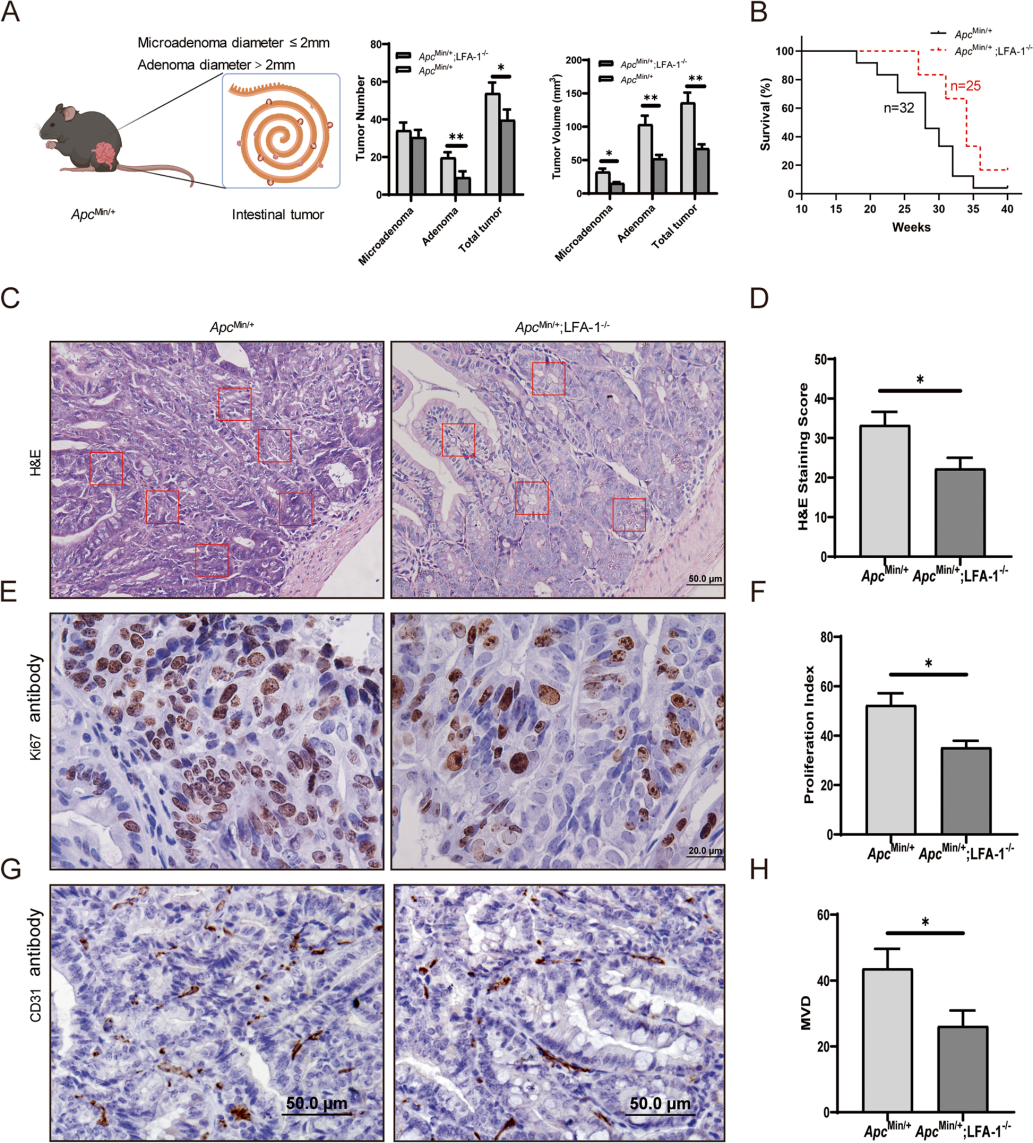
LFA-1 knockout inhibits intestinal tumor growth in *Apc* ^Min/+^;LFA-1^−/−^mice. **A** The number and volume of intestinal microadenoma and adenoma were reduced in *Apc* ^Min/+^;LFA-1^−/−^ mice (*n* = 11) than in *Apc* ^Min/+^ mice (*n* = 10) (**, *P* < 0.01, *P < 0.05, microadenoma diameter ≤ 2 mm; adenoma diameter > 2 mm). **B** The survival rate of *Apc* ^Min/+^;LFA-1^−/−^ mice(*n* = 25) was higher than *Apc* ^Min/+^ mice(*n* = 32) after being assessed by Kaplan-Meier analysis with SPSS software. **C**&**D** H&E staining and scoring of intestinal malignancy in *Apc* ^Min/+^ mice (*n* = 4) and *Apc* ^Min/+^;LFA-1^−/−^ mice (*n* = 4) (The area in the red box is the area of intestinal gland obstruction). **E**&**G** Ki67 and CD31 histochemistry showed the process of tumor proliferation and MVD in *Apc* ^Min/+^ mice (*n* = 4) and *Apc* ^Min/+^;LFA-1^−/−^ mice (*n* = 4). **F** The index of tumor proliferation in *Apc* ^Min/+^ mice (*n* = 4) were slow than *Apc* ^Min/+^;LFA-1^−/−^ mice (*n* = 4, *P < 0.05). H. The MVD in *Apc* ^Min/+^ mice (*n* = 4) were less than *Apc* ^Min/+^;LFA-1^−/−^ mice (*n* = 4, *P < 0.05)

Using another intestinal tumor mice model, we compared the tumor numbers, tumor volumes and survival rates between *Apc* ^Min/+^ mice (*n* = 10) and *Apc* ^Min/+^;LFA-1^−/−^ mice (*n* = 11). The results also showed that the tumor numbers in *Apc* ^Min/+^;LFA-1^−/−^ mice were reduced compared to those in *Apc* ^Min/+^ mice (Fig. 2A, **P < 0.01, *P < 0.05) at 15 weeks,. Especially, the volumes of microadenomas and adenomas were lower in *Apc* ^Min/+^;LFA-1^−/−^ mice than in *Apc* ^Min/+^ mice (Fig. 2A, right; **P < 0.01, *P < 0.05). The survival rate was dramatically increased in *Apc* ^Min/+^;LFA-1^−/−^ mice (Fig. 2B, **P < 0.01).

We also performed pathological IHC staining. The score of the H&E staining showed that LFA-1 knockout slowed down intestinal gland obstruction and alleviated inflammation (Fig. 2C&D, *P < 0.05). Meantime, the growth and infiltration of tumor was assessed using IHC staining, which presented less tumor cell proliferation index Ki67 (Fig. 2E&F, *P < 0.05) and lower microvascular density index CD31 (Fig. 2G&H, *P < 0.05) in *Apc* ^Min/+^;LFA-1^−/−^ mice compared with *Apc* ^Min/+^ mice. These two results suggest that LFA-1 knockout suppresses both endogenous and graft tumor growth.

### The numbers of Treg cells were decreased in LFA-1^−/−^ mice

**Fig. 3:**
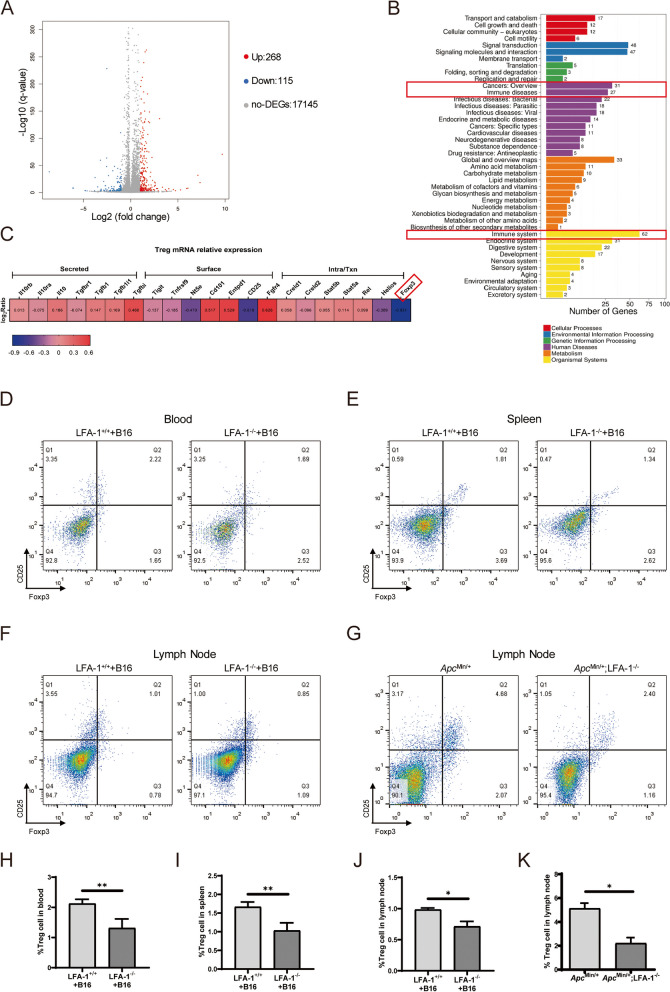
LFA-1 knockout down-regulated Treg cells. **A** The results of spleen mRNA sequencing showed that LFA-1 knockout up-regulated 268 genes and down-regulated 115 genes. **B** Sequencing results showed 31 genes related to cancer overview, 27 genes related to immune diseases, and 62 genes related to the immune system. **C** The Treg cell-related genes were analyzed by spleen mRNA sequencing, and the change of Foxp3 was significant (*n* = 4). **D**-**G** The representative image of Treg flow results in blood, spleen, and mesenteric lymph nodes in LFA-1^−/−^ + B16 mice (*n* = 5) and LFA-1^+/+^ + B16 mice (*n* = 5). Treg cells in mesenteric lymph nodes in *Apc* ^Min/+^ mice (*n* = 4) were lower than *Apc* ^Min/+^;LFA-1^−/−^ mice (*n* = 4). **H**-**J** The percentage of Treg cells in blood, spleen, and mesenteric lymph nodes of LFA-1^−/−^ + B16 mice and LFA-1^+/+^ +B16 mice (*n* = 5, *P < 0.05, **P < 0.01).The number of Treg cells in the mesenteric lymph nodes of *Apc* ^Min/+^;LFA-1^−/−^ mice was measured by flow cytometry and counted (*n* = 4, *P < 0.05)

The above results showed that LFA-1 knockout suppressed tumor growth. To analyze the role of LFA-1 in the tumor immune response, sequence analysis of spleen mRNA showed that LFA-1 knockout resulted in upregulation of 268 genes and downregulation of 115 genes, most of which were related to immune system diseases and tumor immune (Fig. 3A&B). T-lymphocyte is an important component of immune cells. We analyzed the T-lymphocyte subtype-related markers of immune cells including Th1, Th2, Th17 and Thf cells, some of which were up-regulated and some were down-regulated (data not shown). Meanwhile the markers related to Treg cell, including secretory factors, cell membrane genes, and nuclear transcription factor genes, are out of balance. Foxp3 is the most critical transcription factor in Treg cells. By analyzing the value of log_2_Ratio (LFA-1^−/−^/Control), it was found that the expression level of Foxp3 mRNA was significantly down-regulated (Fig. 3C).

To further confirm that Foxp3 expression was altered in the spleens of mice, we performed IHC staining using Foxp3 antibody on spleen tissue from LFA-1^−/−^ mice. Compared to LFA-1^+/+^ mice, the distribution of Foxp3-positive cells in the spleens of LFA-1^−/−^ mice was lower in the white pulp splenic corpuscle (Supplementary Fig. 2A&C, *P < 0.05). And we further stained the spleen tissue of subcutaneous tumor-bearing mice with a Foxp3 antibody. The results also showed that the expression of Foxp3 was less in the white pulp of the spleen of LFA-1^−/−^ tumor-bearing mice (Supplementary Fig. 2B&D, *P < 0.05).

Treg cells can inactivate or regulate T cells, including CD4 + T and CD8 + T cells [21–23]. Circulating CD4 + T and CD8 + T lymphocytes in the blood play an essential role in immune function, and LFA-1 is a key regulator of these functions of T cells. Treg cells were further detected by FCM with anti-Foxp3 and anti-CD25 antibodies and gating on naive CD4 cells. First, we determined the numbers of Treg cells in the blood, spleens and lymph nodes of non-tumor-bearing mice by FCM. The results showed that the decrease of CD4 + CD25 + Foxp3 + Treg cells was significantly different in the blood and lymph nodes (Supplementary Fig. 2E-J; **P < 0.01, *P < 0.05). We also detected the changes in Treg cells in the subcutaneous tumor-bearing model, and the results showed that LFA-1 knockout decreased the numbers of CD4 + CD25 + Foxp3 + Treg cells in the blood, spleens, and lymph nodes (Fig. 3D-F H-J; **P < 0.01, *P < 0.05). There was a significant decrease in the number of Treg cells in tumor-bearing LFA^−/−^ mice.

In addition, we counted CD4 + CD25 + Foxp3 + Treg cells in *Apc* ^Min/+^ mice and *Apc* ^Min/+^;LFA-1^−/−^ mice. The results showed that the percentage of CD4 + CD25 + Foxp3 + Treg cells was decreased in mesenteric lymph nodes in *Apc* ^Min/+^;LFA-1^−/−^ mice (Fig. 3G&K, *P < 0.05). The absolute numbers of Treg cells were also consistent with the percentages of Treg cells (data not shown). The cytokines IL-10, IL-17, and IFN-γ are the key mediators involved in suppressing the growth of tumor cells, IFN-γ, IL-10, and IL-17 production was increased in the spleen of LFA-1-deficient mice, suggesting that tumor immunity was unbalance or enhanced [24]. We also found that the expression levels of IFN-γ and IL-17 in blood of *Apc* ^Min/+^;LFA-1^−/−^ mice increased, and the expression levels of IFN-γ, IL-10, and IL-17 in spleen of *Apc* ^Min/+^;LFA-1^−/−^ mice increased compared to *Apc* ^Min/+^ mice, but there was no premonitory increase in mesentery lymph nodes(Supplementary Fig. 1C-E, *P < 0.05, **P < 0.01). In short, LFA-1 gene knockout affected the expression of Treg cells gene, reduced the number of Treg cells and changed the expression level of some cytokines.

### LFA-1 inhibitor suppressed tumor growth and decreased the numbers of Treg cells

**Fig. 4:**
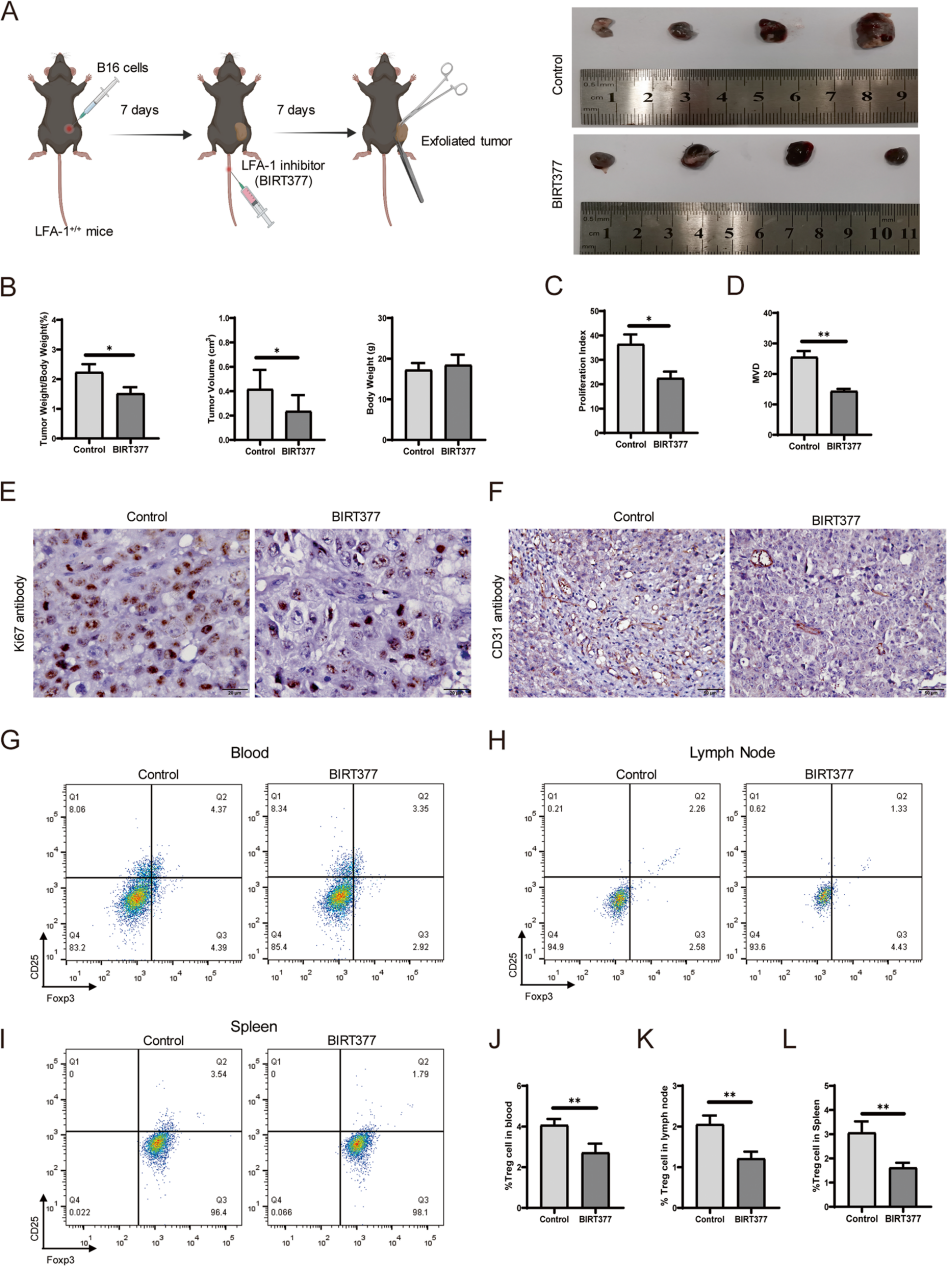
BIRT377 treatment suppressed tumor growth and down-regulate Treg in the spleen. **A** The administration of BIRT377 inhibits the growth of subcutaneous tumors. **B** The subcutaneous tumor weight/bodyweight of the BIRT377 group (*n* = 10) and control group (*n* = 8, *P < 0.05), and the tumor volume of the BIRT377 group (*n* = 10) were smaller than that of control (*n* = 8, *P < 0.05), but the body weight of the BIRT377 group (*n* = 13) compare to control (*n* = 10), it is no statistical difference. **C**&**E** Ki67 antibody used to IHC showed that the tumor proliferation in the BIRT377 group was slow (*n* = 3, *P < 0.05). **D**&**F** CD31 antibody staining showed decreased MVD in the BIRT377 group (*n* = 3, **P < 0.01). **G**&**J** Treg cells determined by flow and the percentage of Treg cells in the blood in the BIRT377 group (*n* = 4) and Control (*n* = 4, **P < 0.01). H&K. the percentage of Treg cells in the mesenteric lymph nodes in the BIRT377 group (*n* = 3) and control group (*n* = 4, **P < 0.01). I&L. The percentage of Treg cells in spleens in the BIRT377 group (*n* = 3) and control group (*n* = 3, **P < 0.01)

BIRT377 is a potent negative allosteric modulator of LFA-1 that binds to the I-domain of LFA-1 as an LFA-1 inhibitor. It has been shown to reduce CD3 + T cells aggregation [19] and can promote degranulation of NK cells [25]. To investigate the role of LFA-1 inhibition in tumor development, mice were treated with BIRT377, and the solvent was used as the control. The results showed the tumor growth was significantly inhibited in the BIRT377 treated group (Fig. 4A) and that the ratio of tumor weight to body weight was significantly decreased (Fig. 4B left, *P < 0.05); in addition, the tumor volume showed a decreasing trend (Fig. 4B middle, *P < 0.05), and the body weights of the mice did not appear in the BIRT377 treated group (*n* = 13) than the control group (*n* = 10; Fig. 4B, right). The drug did not cause other damage to mice, and there were no changes in fur color, food intake or other vital signs. Furthermore, we examined cell proliferation and the microvascular density index in tumor tissues. The results showed that cell proliferation and the microvascular density were lower in the BIRT377 treated group (*n* = 3) than in the control group (*n* = 3; Fig. 4C-F; *P < 0.05, **P < 0.01). Next, we detected Treg cells in the peripheral blood, mesenteric lymph nodes and spleen by flow cytometry and found that the numbers of Treg cells were significantly decreased in the BIRT377 treated group (*n* = 5) compared with the control group (*n* = 4) (Fig. 4G-L, **P < 0.01). Therefore, our data suggest that BIRT377 blocks LFA-1, decreases the Treg cells population, and inhibits mouse tumor growth.

### LFA-1 and Foxp3 are highly correlated in skin cutaneous melanoma and other tumor patients

**Fig. 5:**
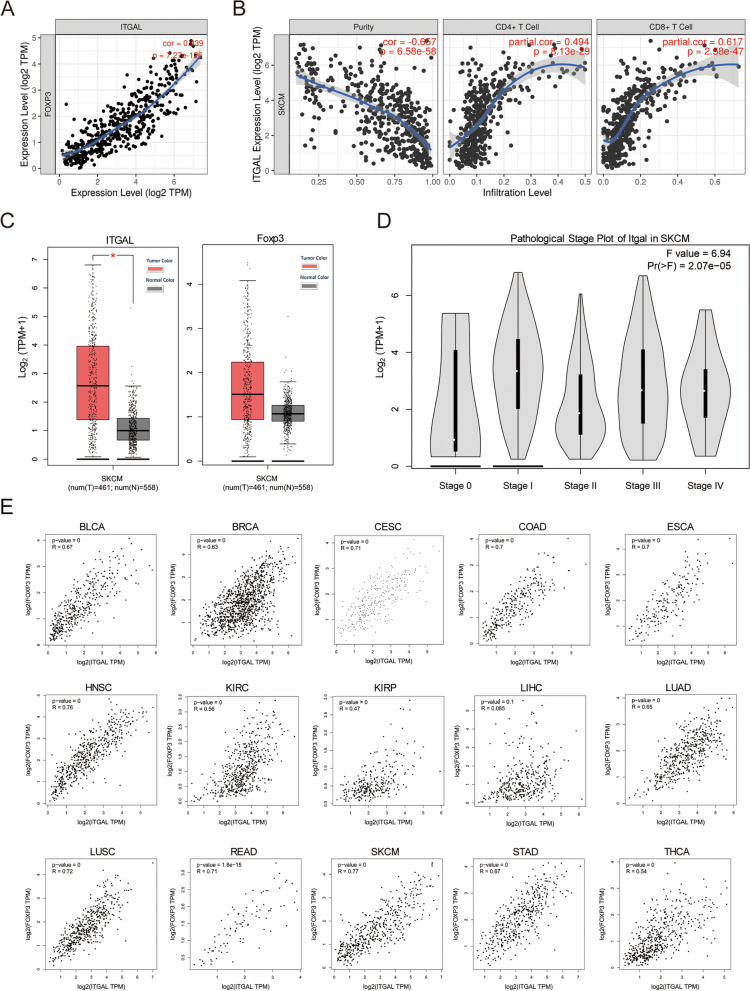
LFA-1 and Foxp3 are highly correlated in SKCM. **A** The correlation between the expression levels of LFA-1 and Foxp3 in SKCM obtained by using TIMER database (https://cistrome.shinyapps.io/timer/). **B** The Infiltration levels of CD4 + T cells and CD8 + T cells in SKCM was analyzed by using the TIMER database (it is the proportion of cancer cells in the admixture. The gene expression levels against tumor purity are always displayed on the left-most panel. Because genes highly expressed in the microenvironment are expected to have negative associations with tumor purity, while the opposite is expected for genes highly expressed in the tumor cells). **C** The expression levels of LFA-1 and Foxp3 in SKCM and normal tissues were analyzed by GEPIA database (http://gepia.cancer-pku.cn/). **D** The expression levels of LFA-1 in SKCM in different pathological stages were analyzed by GEPIA database. **E** The expression of LFA-1 is highly correlated with Foxp3 in Bladder Cancer (BLCA), Breast Cancer (BRCA), Cervical Cancer (CESC), Colon Cancer (COAD), Esophageal Cancer (ESCA), Head and Neck Cancer (HNSC), Kidney Clear Cell Carcinoma (KIRC), Kidney Papillary Cell Carcinoma (KIRP), Liver Cancer (LIHC), Lung Adenocarcinoma (LUAD), Lung Squamous Cell Carcinoma (LUSC), Rectal Cancer (READ), Melanoma (SKCM), Stomach Cancer (STAD), Thyroid Cancer (THCA) tumors. These data were obtained from GEPIA (http://gepia.cancer-pku.cn/)

**Fig. 6:**
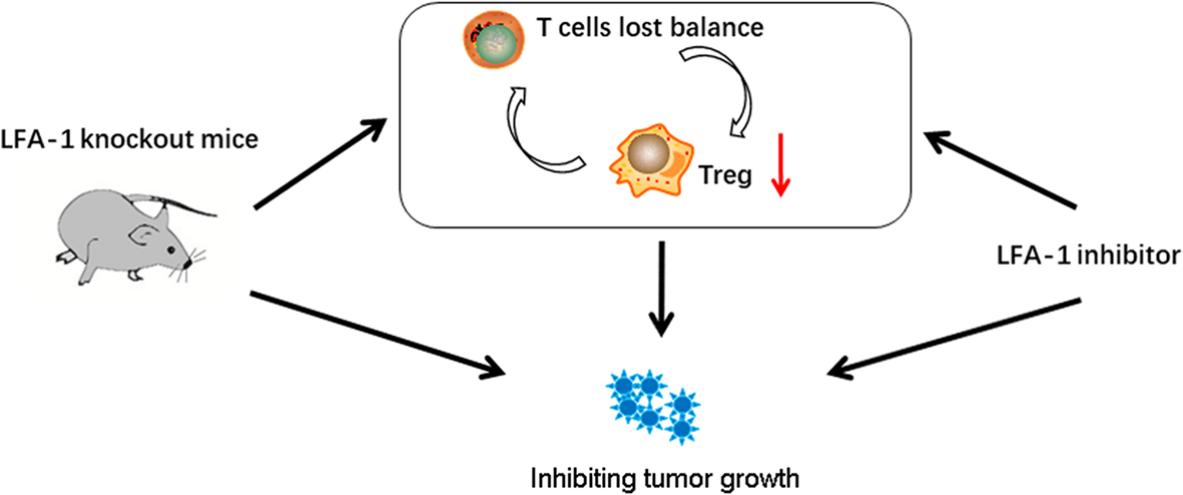
LFA-1 inhibitors, like LFA-1 knockout, inhibit tumor growth and are associated with decreased Treg cells

The above results suggested that LFA-1 (Itgal) knockout would suppress tumor growth and decrease Treg cell numbers. Next, we analyze the correlation between LFA-1 and Treg cells specific nuclear transcription factor (Foxp3) in skin cutaneous melanoma(SKCM) and immune cell infiltration in the TIMER tumor database. The results showed that LFA-1 was highly correlated with Foxp3 in SKCM (Fig. 5A), and the LFA-1 expression level is negatively associated with tumor cells purity and positively associated with the infiltration of CD4 + T cells and CD8 + T cells (Fig. 5B). Tumour purity is the proportion of cancer cells in the admixture [26]. At the same time, we used the GEPIA database to analyze the expression level of LFA-1 and Foxp3 in SKCM and in different clinic stages of SKCM. The results showed that LFA-1 and Foxp3 were both highly expressed in SKCM, the expression level of LFA-1 in tumor was high than normal tissues, and the expression was the highest in Stage I of SKCM (Fig. 5C&D). In addition, in patients with many cancer types, like Bladder Cancer (BLCA), Breast Cancer (BRCA), Cervical Cancer (CESC), Colon Cancer (COAD), Esophageal Cancer (ESCA), Head and Neck Cancer (HNSC), Kidney Clear Cell Carcinoma (KIRC), Kidney Papillary Cell Carcinoma (KIRP), Liver Cancer (LIHC), Lung Adenocarcinoma (LUAD), Lung Squamous Cell Carcinoma (LUSC), Rectal Cancer (READ), Melanoma (SKCM), Stomach Cancer (STAD), Thyroid Cancer (THCA), the LFA-1 expression is positively correlatedwith foxp3 in tumor tissue (Fig. 5E). The mRNA sequencing analysis obtained from clinical tumor databases are consistent with our results and support our data that LFA-1 is associated with Treg cells in tumor tissue (Fig. 6).

## Discussion

Previous studies have shown that LFA-1 deficiency can produce a normal CTL response to viral infection but fails to reject immunogenic tumors [6]. Here, LFA-1 knockout suppressed the growth of intestinal tumors in *Apc* ^Min/+^ mice and also inhibited subcutaneous tumor growth in mouse model,

simultaneously accompanied by a decrease in the number of Treg cells.

LFA-1 is regarded as a bidirectional signaling molecule that controls cytoskeleton-dependent T-cell activation and is not constitutively active until it binds to ICAM-1 [27, 28]. LFA-1 play an important role in specific immune suppression and tolerance, initial LFA-1-dependent formation of Treg aggregates on immature Dendritic cells (DCs) and subsequent LFA-1- and CTLA-4-dependent active down-modulation of CD80/86 expression on DCs [29]. Treg cells have a crucial role in the immune system by preventing autoimmunity and limiting immunopathology. Importantly, they constitute a major barrier to effective antitumor immunity and sterilizing immunity to chronic viral infections [8]. Treg cells were impaired in LFA-1^−/−^ mice, as confirmed in previous studies [5, 29–31]. Our results indicated an abnormal number of Treg cells in the mesenteric lymph nodes of LFA-1^−/−^ mice. David P. et al. proved that LFA-1 regulates the transport of lymphocytes to peripheral lymph nodes and to a lesser extent to mesenteric lymph nodes and acute inflammatory sites [32]. LFA-1 is required for optimal CD4 + T cell priming in vivo [33]. However, CD4 + T cell upregulation is insufficient for antitumor effects [34]. Overall, these findings support our results that the number of Treg cells is decreased in LFA-1^−/−^ mice, but the mechanism remains unclear.

Moreover, the analysis of the GEPIA database showed that the expression levels of LFA-1 and the Treg cell transcription factor Foxp3 are interrelated in numerous tumors in our study. Still, other immune cells cannot be excluded from participating in the immune response during tumor growth. In addition, another study showed that blockade of CD18 (an LFA-1 subtype) can impair melanoma cell transmigration to control the metastatic mechanism in melanoma [35], which also supported our results.

LFA-1 inhibitors have been reported; BIRT377, a member of a novel class of low-molecular-weight hydantoins, is the first small molecule inhibitor of LFA-1 and may have potential as a therapeutic agent [36, 37]. BIRT377 has been shown to block the binding of LFA-1/ICAM-1 and inhibit the interaction between T cells and antigen-presenting cells [19, 36, 38]. Blocking LFA-1 in the spinal cord with BIRT377 can eliminate allodynia in prenatal alcohol exposure (PAE) rats with sciatic neuropathy (CCI) for 10 or 28 days. BIRT377 can antagonize the effect of LFA-1 on reducing peripheral cell migration and may directly change the inflammatory bias of cells [20, 39, 40]. In our study, we showed that BIRT377, retard the growth of subcutaneous tumors and decrease the number of Treg cells.

LFA-1 deficiency is in all cell of the body and that its inhibitors are active on every cells bearing the target. In order to compare the immune function of LFA-1 negative Treg cells and wild-type Treg cells, we transplanted the bone marrow of LFA-1^−/−^ mice and C57 mice into Rag-1^−/−^ mice by tail vein surgery. Rag-1^−/−^ mice showed severe early development retardation of T/B cells. we transplanted bone marrow of LFA-1^−/−^ mice through tail vein into Rag-1^−/−^ mice (4 mice), and bone marrow of C57 mice as control transplanting into another group of Rag-1^−/−^ mice (4 mice). During the first week after transplantation, both groups of mice were well. But one week later, two Rag-1^−/−^ mice transplanted with bone marrow of C57 mice died. By the third week, all the mice in this group were dead. However, these 4 mice that received bone marrow transplantation from LFA-1^−/−^ mice stayed alive (Supplementary Fig. 3). This data demonstrates that LFA-1 deletion contributed to prolonging the survival rate of Rag-1^−/−^ mice after bone marrow transplant. the reason keeps unknow. It is suggested that LFA-1 plays an important role in immune reaction. Indeed, so far, the limitation of the study is that it is difficult to neutralize and depleted Treg cells to confirm the effects and function of Treg cells in LFA-1^−/−^ mice.

In conclusion, our findings reveal the new role of LFA-1 in tumors growth in mice is that the deletion of LFA-1 inhibits tumor growth. In addition, the decrease of Treg cells and the changes of related cytokines may help to inhibit tumor growth. It speculates that LFA-1 is a potential and promising biomarker for evaluating tumor growth and a novel target for tumor immunotherapy.

### Supplementary Information

**Additional file 1.**

## Data Availability

The datasets used and/or analyzed during the current study are available from the corresponding author on reasonable request.

## Abbreviations

LFA-1: Lymphocyte function-associated antigen-1
Treg: Regulatory T cells
EAE: Experimental autoimmune encephalomyelitis
CTL: Cytoxic T-cell lymphocyte
ICAM-1: Intercellular adhesion molecule 1
H&E: Hematoxylin-Eosin staining
IHC: Immunohistochemistry
DAB: Diaminobenzidine
MVD: Microvascular density
FCM: Flow cytometry analysis
PBS: Phosphate-buffered saline
FBS: Fetal bovine serum
GAPDH: Glyceraldehyde 3-phosphate dehydrogenase
qRT-PCR: Quantitative reverse transcription-polymerase chain reaction
SKCM: Skin cutaneous melanoma
