# Supplementary material for: LFA-1 knockout inhibited the tumor growth and is correlated with treg cells
Source: Cell Commun Signal. 2023 Sep 18;21:233. doi: 10.1186/s12964-023-01238-6 (PMC10506322; doi:10.1186/s12964-023-01238-6)
Supplement: Supplementary file 2 — Additional file 1. [file 12964_2023_1238_MOESM1_ESM.docx]

**Supplemental data**

| Table. 1 qPCR primers (mouse) | | |
| --- | --- | --- |
| Target Gene | Forward (5'‑3') | Reverse (5'‑3') |
| IFN-γ | TGAACGCTACACACTGCATCTTGG | CGACTCCTTTTCCGCTTCCTGAG |
| IL-10 | AGCCTTATCGGAAATGATCCAGT | GGCCTTGTAGACACCTTGGT |
| IL-17 | CACCCAGAGCACCAGCTGAT | AATCAATAGCACGAACTG |
| IL-2 | GACACTTGTGCTCCTTGTCA | TCAATTCTGTGGCCTGCTTG |
| TNF-α | GGAACACGTCGTGGGATAATG | GGCAGACTTTGGATGCTTCTT |
| GAPDH | GGTGAAGGTCGGTGTGAACG | CTCGCTCCTGGAAGATGGTG |

**
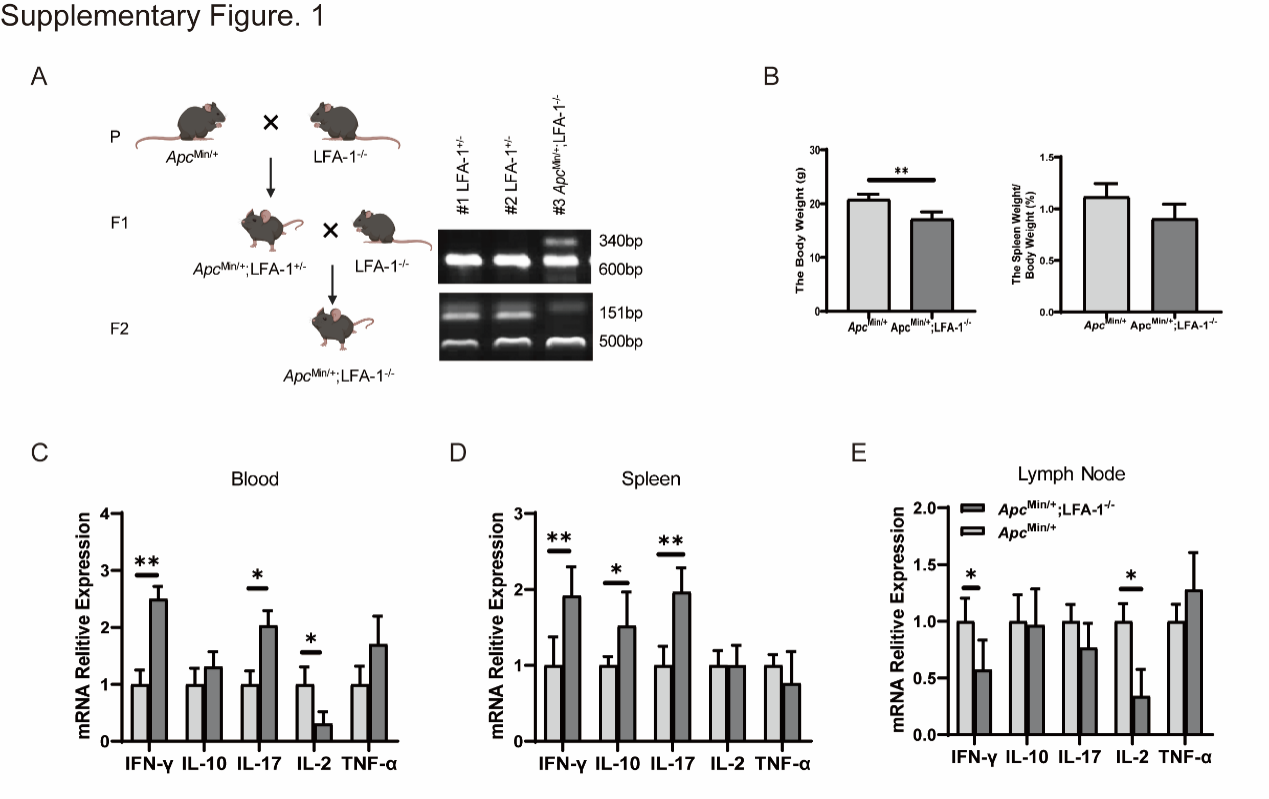
**

**S. Figure. 1 Disorder of inflammatory cytokines in *Apc*^Min/+^;LFA-1^-/-^ mice**

A. *Apc*^Min/+^;LFA-1^-/-^ mice were obtained by cross-breeding *Apc*^Min/+^ mice with LFA-1^-/-^ mice and the genotype was identified by PCR, the representative image of gel electrophoresis showing the genotype of the mice was *Apc*^Min/+^;LFA-1^-/-^mice (#3). B. Compared with *Apc*^Min/+^ mice (n=12), *Apc*^Min/+^;LFA-1^-/-^ mice (n=10) lost more weight (***P*<0.01); but there is no difference in the percentage of spleen weight to body weight. C-E: Relative mRNA expression levels of IFN-γ, IL-10, IL-17, IL-2 and TNF-α in blood, spleen and lymph nodes (n=5, **P*<0.05, ***P*<0.01).


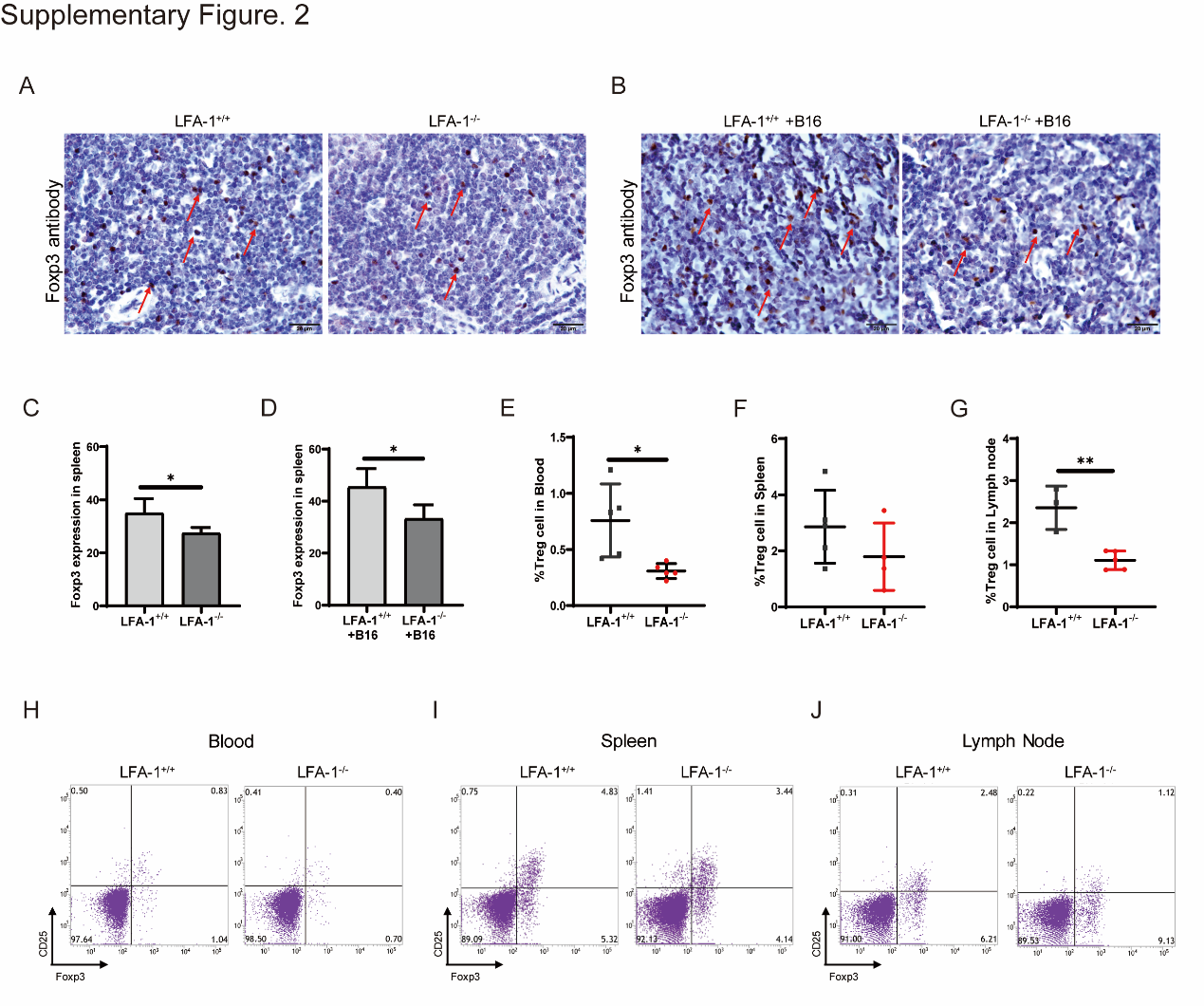


**Supplementary Figure. 2 The distribution of Foxp3 expression in spleen**

A&C. The distribution of Foxp3+ cells in the spleen of LFA-1^-/-^mice (n=3) compare with LFA-1^+/+^ mice (n=3, **P*<0.05). B&D. The distribution of Foxp3 in the spleen of LFA-1^-/-^ + B16 mice(n=3) compare with LFA-1^+/+^+ B16 mice (n=3, **P*<0.05). E&H. Treg cells flow results and the percentage of Treg cells in the blood of LFA-1^-/-^ mice (n=5) and LFA-1^+/+^mice (n=5, **P*<0.05). F&I. The percentage of Treg cells in the spleen of LFA-1^-/-^ mice (n=4) and LFA-1^+/+^mice (n=5). G&J. The percentage of Treg cells in mesenteric lymph nodes of LFA-1^-/-^mice (n=5) and LFA-1^+/+^mice (n=3, **P*<0.05).

**Supplementary Figure. 3**


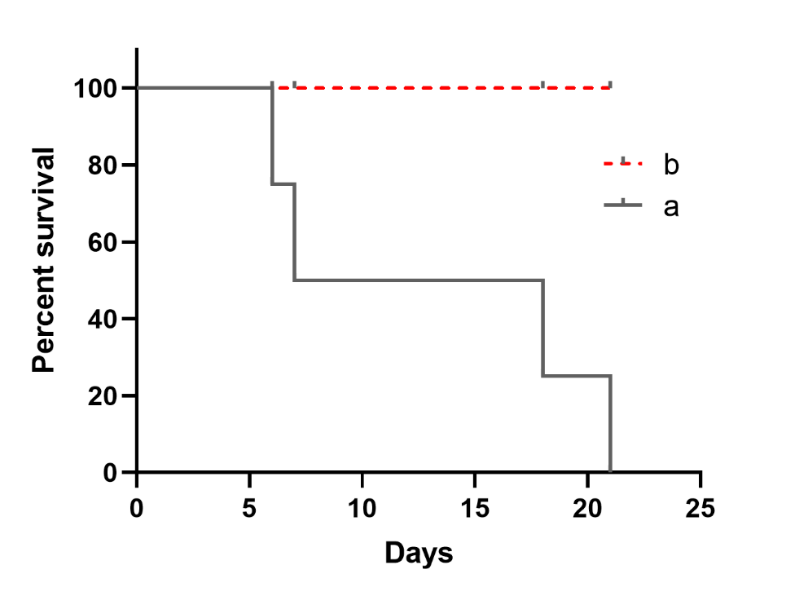


**Supplementary Figure. 3 Survival rate of Rag-1^-/-^ mice after bone marrow transplantation**

1. “a” line indicated that C57 mice bone marrow being transplanted to Rag-1^-/-^ mice (n=4).

“b” red line indicated that LFA-1^-/-^ mice bone marrow being transplanted to Rag-1^-/-^ mice (n=4).

**Supplementary data for Figure 5.**

**We used TIMER (https://cistrome.shinyapps.io/timer/) and GEPIA (http://gepia.cancer-pku.cn/) online database platform to analyze and make all the images of Fig4.**

**A:** open “TIMER” → ‘Correlation’, (<https://cistrome.shinyapps.io/timer/>)

Y-axis: ITGAL

X-axis: Foxp3

Correlation Adjusted by: no

Submit

**B:** open “TIMER” → ‘Gene’, (<https://cistrome.shinyapps.io/timer/>)

Gene Symbol: ITGAL

Cancer Types: SKCM (Skin Cutaneous Melanoma)

Immune infiltrates: CD4+T Cell and CD8+T Cell

Submit

**C:** open “GEPIA” → ‘Expression DIY’ → ‘Boxplot’

Gene: ITGAL or Foxp3

Cancer name: SKCM

Plot ([http://gepia.cancer-pku.cn/detail.php?clicktag=matrix###](http://gepia.cancer-pku.cn/detail.php?clicktag=matrix))

**D:** open “GEPIA” → ‘Expression DIY’ → ‘Stage plot’

Gene: Itgal

Cancer name: SKCM

Plot ([http://gepia.cancer-pku.cn/detail.php?clicktag=matrix###](http://gepia.cancer-pku.cn/detail.php?clicktag=matrix))

**E:** open “GEPIA” → ‘Correlation’ ([http://gepia.cancer-pku.cn/detail.php?clicktag=matrix###](http://gepia.cancer-pku.cn/detail.php?clicktag=matrix))

Gene A: ITGAL

Gene B: Fopx3

TCGA Tumor (Cancer name): add separately ‘BLCA, BRCA, CESC, COAD, ESCA, HNSC, KIRC, KIPP, LIHC, LUAD, LUSC, READ, SKCM, STAD, THCA’

Plot separately
